# Cardiac Murmurs Iu Chorea

**Published:** 1871-02

**Authors:** 


Cardiac Murmurs in Chorea.—The murmur which is not unfrequently heard in choreic patients, Sir Wm. Jenner affirms is a mitral regurgitant murmur, due to irregular action of the papilary muscles, sometimes accompanied by irregular contraction of the heart itself. Thus cases are met in which there is irregular action of the heart with an occasional murmur, or irregular action with a constant murmur, or regular cardiac action either with a constant or inconstant murmur; and in all these cases the murmur, and irregular action when present, disappear either shortly before or shortly after the cessation of the choreic movements of the voluntary muscles. Murmurs first detected during a choreic attack may remain after its subsidence, but in these cases there has been at some time inflammation of the endocardium.

